# Dr. Worp Van Peyma

**Published:** 1910-09

**Authors:** 


Dr. Worp Van Peyma, of Buffalo, died in this city August 19, 1910. He practised for many years at Clarence Center, N. Y., but came to Buffalo about twenty years ago where he resided until his death.

